# Toward Improving Triplet Energy Transfer from Tetracene to Silicon Using a Covalently Bound Tetracene Seed Layer

**DOI:** 10.1021/acs.jpclett.3c00589

**Published:** 2023-05-08

**Authors:** Alyssa F. J. van den Boom, Silvia Ferro, María Gelvez-Rueda, Han Zuilhof, Bruno Ehrler

**Affiliations:** † Laboratory of Organic Chemistry, Wageningen University, 6708 WE Wageningen, The Netherlands; ‡ Center for Nanophotonics, AMOLF, Science Park 104, 1098 XG Amsterdam, The Netherlands; § School of Pharmaceutical Science and Technology, Tianjin University, Tianjin 300072, China

## Abstract

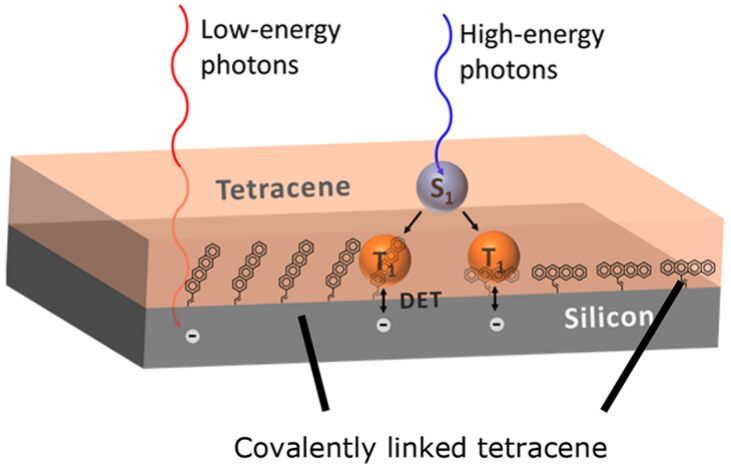

Silicon solar cells are operating close to the theoretical maximum efficiency limit. To increase their efficiency beyond this limit, it is necessary to decrease energy losses occurring for high-energy photons. A sensitizing layer of singlet-fission material can in principle double the current generated by high-energy photons, and significantly reduce energy losses from high-energy photons within the solar cell. Here, we construct a model of such a solar cell, using Si(111) surfaces and tetracene. To increase the energy transfer between the two layers, a series of tetracene derivatives was synthesized, and the molecules were covalently attached onto the silicon surface as a seed layer. Using X-ray diffraction, a shift in crystal structure and ordering of the tetracene close to the seed layer can be observed. Unfortunately, the effect on the energy transfer was limited, showing a need for further investigations into the effect of the seed layer.

Solar energy conversion plays an important role in the development toward 100% sustainable energy production. Up to now, the main way of converting solar energy into electricity is by using photovoltaic solar panels. These panels are generally made of silicon, due to its wide availability, its established place in the semiconductor industry, the resulting relatively low cost of highly pure material, and because, theoretically speaking, silicon has one of the most optimal bandgaps for solar energy conversion, as calculated first in 1961.^1−3^ Yet even with an almost ideal bandgap, the maximum conversion for silicon is still limited to 29% of all available solar energy.^4^ This is mostly a result of the fixed bandgap of silicon: photons with an energy higher than the bandgap, meaning all photons with a wavelength shorter than ∼1100 nm, lose their energy in excess of the bandgap of silicon to thermal losses after absorption, resulting in highly inefficient use of the high-energy part of the solar spectrum. It is therefore not surprising that much research has been performed on improving the efficiency with which these high-energy photons are absorbed and converted into electricity, as a means of boosting solar cell performance. This has e.g. been done by the development of tandem solar cells, in which the high-energy photons are absorbed by a material with a high bandgap, stacked on top of a low-bandgap material to absorb the low-energy photons.^5^ The commercially most promising tandem cells are made, in part, of perovskites, as these materials have easily tunable bandgaps, and are easy to process. However, perovskites suffer from low stability, decreasing the lifetime of the overall tandem cell.^6^ Moreover, when the two subcells are connected in series, a tandem cell requires current-matching between them, which is technically challenging, and to some extent dependent on the incident lighting conditions.^5,7^

Another promising approach to make better use of the high-energy light is to use sensitizing layers on top of conventional single-junction solar cells. These sensitizing layers are ideally made from materials that can undergo a carrier multiplication process, for example singlet fission. Such a layer can, in principle, double the current coming from high-energy photons.^9,10^ The singlet fission process itself can be roughly described by two steps:^11,12^ first, a high-energy photon is absorbed by one molecule to create an excited singlet exciton state; then, the excited singlet exciton converts to a lower-energy triplet exciton state, while the excess energy is simultaneously transferred to an adjacent molecule and used to excite this second molecule into the triplet state as well. After this process, the two molecules both carry an excited triplet exciton state. Due to the nature of the triplet state, recombining to the ground state is formally spin-forbidden, which in practice means these excited states decay relatively slowly. Yet, the energy of these excited systems can be extracted using a suitable semiconductor, to yield two electron–hole pairs with about half the energy of the original photon. Using this solar cell design with a silicon base cell, an increase in maximum solar cell efficiency from 29% to 35% can be achieved, depending on the materials and triplet exciton transfer process used (Figure 1).^13^ Currently, one of the key bottlenecks to make a singlet fission-sensitized silicon solar cell a reality is the transfer of the triplet exciton state into silicon. There have even been previous examples of silicon solar cells with a tetracene sensitizing layer, yet the efficiency of these cells was either not increased, or only very little, and interfacial layers or modification of the silicon surface were needed to facilitate the increase in efficiency.^13−15^ Previous studies have shown that tetracene tends to orient itself almost perpendicular to the silicon surface when deposited.^16^ Such an orientation leads to a low overlap in wave functions between the tetracene and silicon–and therefore a low energy-transfer efficiency. However, when the orientation of the tetracene on the silicon surface is changed to a more parallel orientation, energy transfer to silicon is increased significantly.^17^

**Figure 1:**
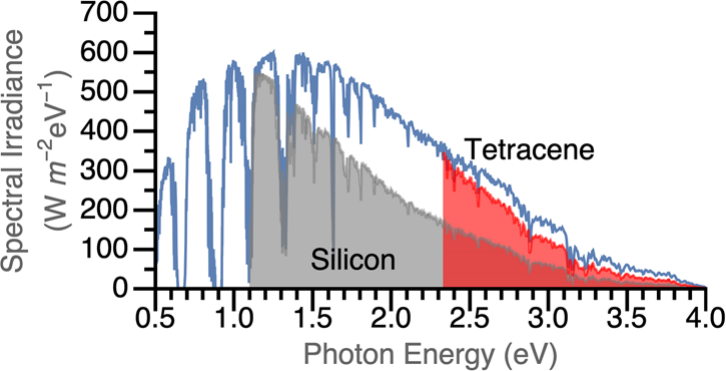
Theoretical maximum energy extracted from the solar spectrum by a silicon solar cell with and without a tetracene sensitizing layer, based solely on the bandgaps involved. Blue curve indicates the AM1.5G solar spectrum. Loss mechanisms besides thermalization losses are disregarded.^8^

In this work, our approach is to covalently attach a seed layer of tetracene derivatives to the silicon surface, and subsequently deposit further layers of tetracene on top of this seed layer. The seed layer serves to orient the deposited tetracene layers for optimal energy transfer. Tetracene has a triplet energy that is almost perfectly matched to the bandgap of silicon,^14^ and furthermore has the benefit that–when applied on top of a silicon solar cell–it lowers the temperature in, and extends the lifetime of the underlying silicon solar cell by reducing heat production due to thermalization losses.^18^ Moreover, tetracene has already been used previously for the sensitization of other materials, such as C_60_^19,20^ (with an interfacial layer of copper phthalocyanine),^21,22^ or PbS nanoparticles,^23,24^ and in all cases showed energy transfer to the underlying substrate.

Two tetracene derivatives were designed for this study: one with a linker on the 5-position (**1**), and one with a linker on the 2-position (**2**, Figure 2). Derivative **1** is expected to have a more favorable orientation when attached to silicon, but the presence of the linker on the 5-position, where the electron density is highest in the LUMO and during excitation to the S_1_ state,^25,26^ implies that the physical and optical properties of this derivative will show a significant shift compared to those of unmodified tetracene. This shift would certainly be larger than the shift expected for **2**,^27^ where the linker is at a position of relatively low electron density, meaning its properties will be largely similar to those of tetracene. However, the orientation on the surface will be more perpendicular compared to derivative **1**, causing the wave function overlap with the silicon surface to be lower.

**Figure 2:**
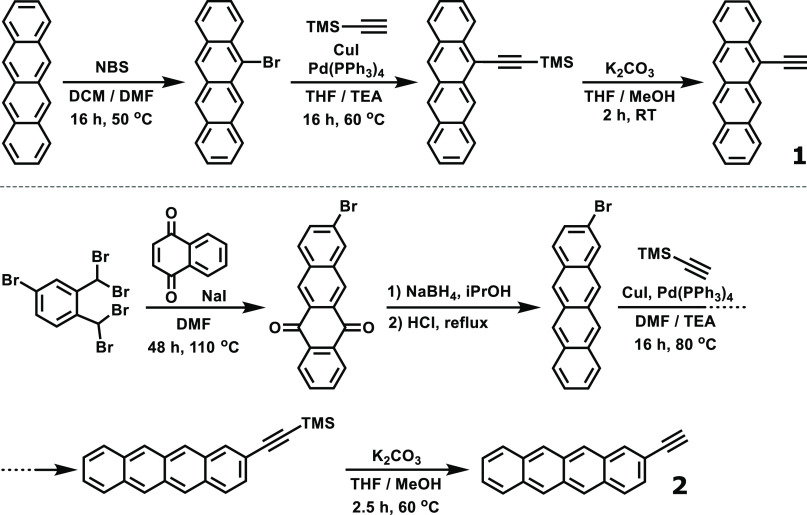
Synthesis schemes for tetracene derivatives **1** and **2**.

The effects of the linker position could already clearly be seen during synthesis: while derivative **1** displayed good solubility, derivative **2** showed an extremely poor solubility in most common organic solvents, in line with the behavior of unfunctionalized tetracene. Still, both derivatives could be obtained on a multiple-gram scale, thereby showing promise for further scale-up. Experimental details for the synthesis and characterization are given in the Supporting Information. When recording the optical properties, using a trimethylsilyl group to protect the highly reactive ethynyl moiety, the differences between **1** and **2** were further highlighted (Figure 3). Here, again, the spectra for derivative **2** more closely resemble the properties of tetracene itself, with three clear vibronic peaks in both the excitation, emission, and absorption spectra, and only a ∼ 10 nm red-shift for all peaks with respect to tetracene (Table 1). In terms of energy, this shift in wavelengths corresponds to a 0.05 eV difference for the excitation energies, and a 0.06 eV difference for the emission energies. For **1**, the shift in excitation and emission energies is larger (0.16 eV for the excitation, and 0.20 eV for the emission), and the peaks themselves are broader compared to both tetracene and **2**. These results for **1** are in line with previous studies on tetracene dimers, where a similar linker structure was used to link two tetracene molecules together,^28,29^ and in line with our assumption that the linker in the 2-position should affect the singlet exciton less strongly than the linker in the 5-position. Previous studies on tetracene molecules containing substituents similar to the ethynyl moiety on the 5-position also confirmed that the singlet fission capabilities of such molecules were intact, both in solution and in thin films.^28,29^ Therefore, the observed shifts in optical properties are not expected to negatively impact the energy transfer in our model system. In fact, the small red-shift observed for especially **2** may even be beneficial, by funneling the triplet energy from the deposited unfunctionalized tetracene layer to the functionalized seed layer and into the final solar cell. Such “cascade” systems using tetracene derivatives have already been proven to increase the efficiency of tetracene-sensitized solar cells.^20^

**Figure 3:**
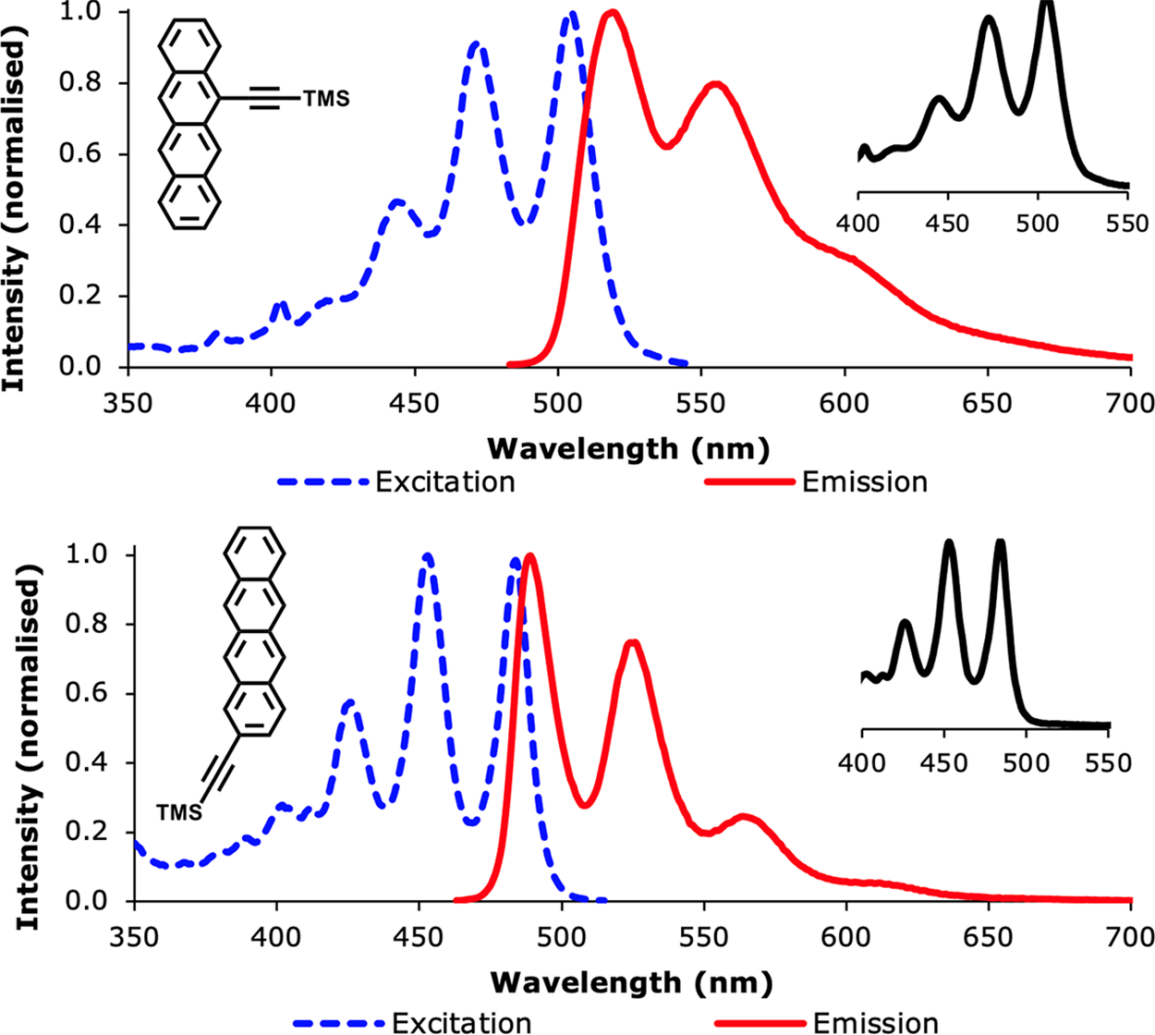
Excitation and emission spectra of trimethylsilyl-protected 1 (top) and 2 (bottom) in dichloromethane. The inserts in the top right corners show the absorption spectra.

**Table 1:** Excitation and Emission Maxima for Tetracene, 5-((Trimethylsilyl)-ethynyl)tetracene (**1-TMS**), and 2-((Trimethylsilyl)ethynyl)tetracene (**2-TMS**)

	Excitation maxima (nm)			Emission maxima (nm)		
Tetracene	419	445	475	478	512	550
**1-TMS**	444	472	504	519	556	605
**2-TMS**	426	453	484	489	526	564

After measuring the properties of both derivatives in solution, we fabricated model devices by functionalizing hydrogen-terminated silicon (111) surfaces (H–Si(111)) with **1** and **2** (Figure 4), which would form the heart of a singlet fission/silicon solar cell. It is for this purpose that an ethynyl linker was used, as this linker has several advantages over other functional groups when it comes to functionalizing H–Si(111):^30^ 1) the ethynyl linker is the smallest linker containing a terminal alkyne, ensuring the distance of the seed layer to the silicon surface is as small as possible; 2) alkynes react faster and under milder conditions to H–Si(111) than other carbon-based linkers;^31^ 3) alkynes provide good packing densities and even monolayers;^32,33^ and 4) after modification, alkenyl-linked monolayers as derived from alkynes show better stability than alkyl-linked monolayers,^34^ likely linked to their higher density on the surface.^35^ Since the tetracene monolayer we attach to Si(111) has some distinct aromatic signals, it should be possible to confirm successful modification using infrared spectroscopy (IR). However, the IR spectrum that was recorded of the functionalized surface showed no significant peaks that could be attributed to an aromatic moiety on the surface (Supporting Information, Figure S1), and could not be used to confidently state successful modification had taken place. Therefore, two fluorinated derivatives of **1** and **2** were prepared (**1F** and **2F**, see Supporting Information Figures S2 and S3), according to the synthesis outlined in Figure 5. After functionalization of fresh surfaces with these fluorinated derivatives, attachment could easily be confirmed using XPS, by looking for the presence of a fluorine peak in the wide scan. Since the surfaces were sonicated in DCM after functionalization, any physisorbed tetracene or tetracene derivative is removed prior to the XPS measurements, ensuring that the fluorine signal observed comes from covalently bound tetracene moieties. To confirm covalent attachment even further, several surfaces were rubbed over fiber-free paper before XPS measurements, and no difference in the fluorine signal was observed between these and only sonicated samples. Due to the similarity between the fluorinated and nonfluorinated tetracene derivatives, we assume successful attachment of the nonfluorinated **1** and **2** occurred to a similar degree as found for **1F** and **2F**.

**Figure 4:**
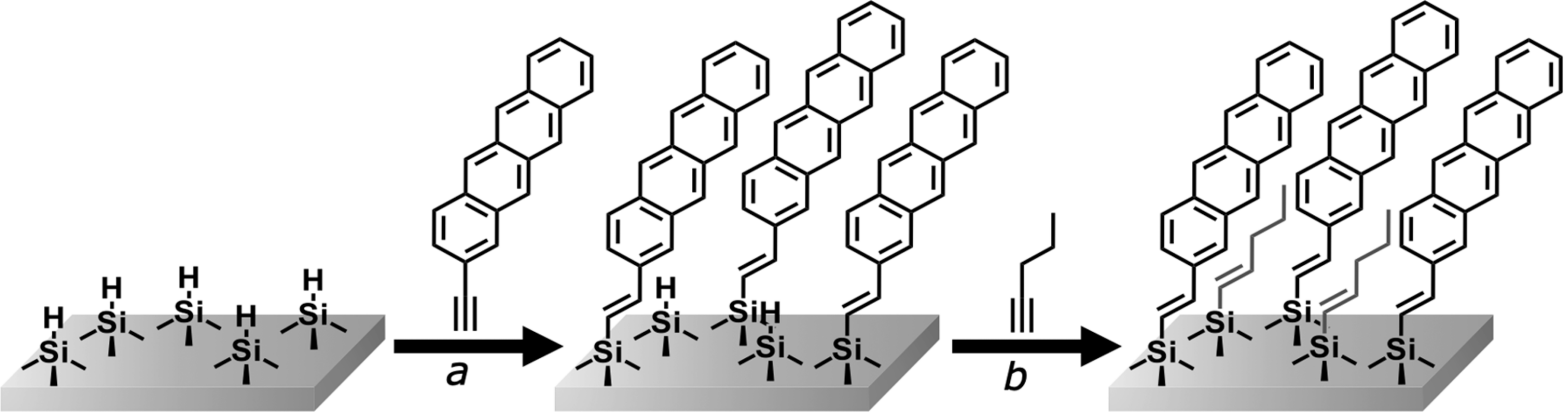
Schematic representation of the surface functionalization reactions. (a) Functionalization with 2-ethynyltetracene; (b) backfilling with 1-pentyne.

**Figure 5:**
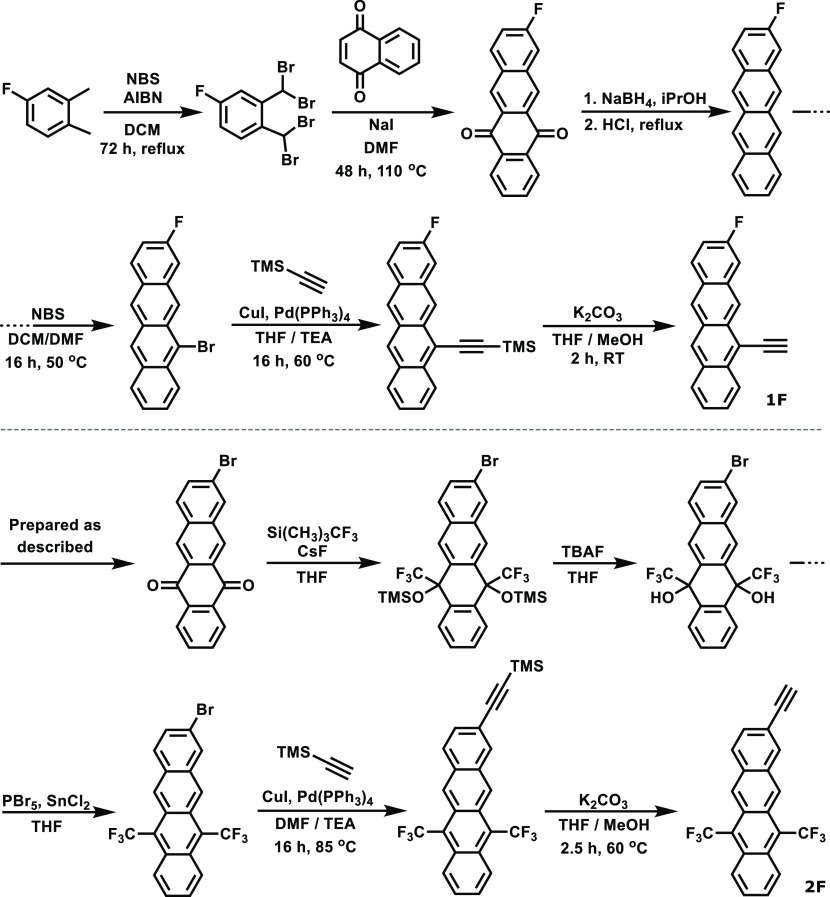
Synthesis schemes for **1F** and **2F**, the fluorinated versions of tetracene derivatives **1** and **2** for XPS analysis.

For surfaces functionalized with **1F**, a ratio of C/F of 21.4:1 is found in the XPS wide scan, close to the expected ratio of 20:1 (Figure 6). The amount of carbon is slightly higher due to a backfilling reaction with 1-pentyne, performed after functionalization with **1F**. This backfilling is done to prevent the oxidation of unfunctionalized Si–H sites, which would lead to the formation of an insulating layer of SiO_*x*_ on the silicon surface, that in turn would reduce the energy transfer efficiency in the final solar cell.^36,37^ Based on the C/F ratio, the final surface is functionalized with a ratio of 4:1 **1F**/1-pentyne. We find relatively stable surface passivation with the pentyne backfilling, after several weeks exposure to ambient atmosphere, the amount of surface oxidation for a backfilled surface is only 3.8% (Figure 6 right), instead of the 4.9% observed for nonbackfilled surfaces (Supporting Information, Figure S4), demonstrating a reduction in oxidation. As expected, full passivation was not achieved, even with backfilling, due to the molecular footprint of **1F**, which apparently limits access to the surface for the 1-pentyne, thus hampering full backfilling. Future work into the use of smaller, gaseous alkynes as backfilling materials would be highly useful to further decrease oxidation.

**Figure 6:**
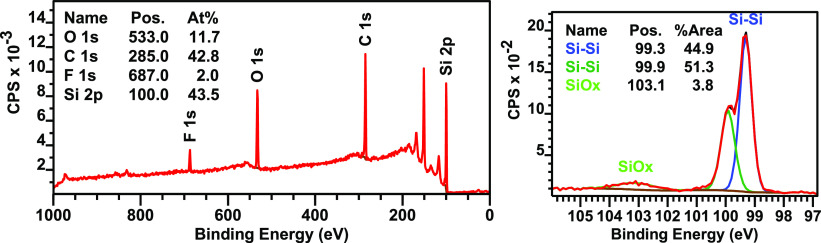
XPS wide (left) and Si 2p narrow (right) scans of a representative surface functionalized with 1F after backfilling with 1-pentyne.

For surfaces functionalized with **2F**, the ratio of C/F is a much higher than the theoretically expected ratio of 22:6 (Figure 7). This is expected, as the two bulky −CF_3_ groups on **2F** likely lead to a low packing density of this derivative on the surface. Still, the more perpendicular orientation leaves more openings for backfilling, resulting in a surface with a ratio of ∼1:8 for **2F**/1-pentyne. The increased amount of backfilling can also be seen in the lower amount of surface oxidation: with backfilling, 2.7% of Si–O_*x*_ is observed after several weeks in ambient atmosphere, while this increases to 7.5% without backfilling. This demonstrates the importance of the backfilling procedure for **2F**-functionalized surfaces, which decreases oxidation by roughly two-thirds.

**Figure 7:**
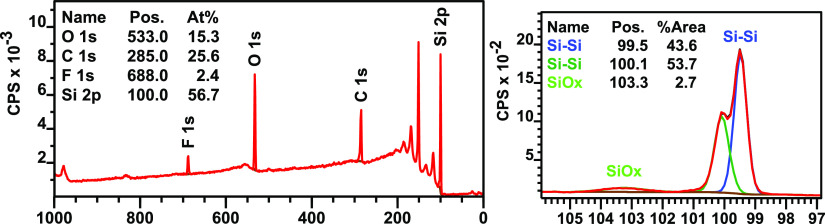
XPS wide (left) and Si 2p narrow (right) scans of a representative surface functionalized with 2F after backfilling with 1-pentyne.

We note that the XPS spectra of the surfaces functionalized with unmodified molecules **1, 2**, and 1-pentyne show no significant F peak (see Supporting Information, Figures S5), which shows that the other processing steps such as the NH_4_F etching do not leave fluorine on the silicon surface.

After confirming the presence of a covalently bound seed layer of **1** or **2**, a 100 nm layer of underivatized tetracene was deposited on silicon surfaces functionalized with **1** and **2**, as well as on a surface functionalized with only 1-pentyne, used as a control sample. The crystallinity of the deposited layers was measured with XRD, and an interesting result was found; where surfaces functionalized with **2** showed good crystallinity of the tetracene layer, surfaces functionalized with **1** showed little to no crystallinity (Figure 8). In fact, the crystallinity of these last surfaces was even lower than that of the reference sample functionalized with only 1-pentyne. Perhaps the tetracene deposited on the surface functionalized with **1** cannot interact properly with the covalently bound tetracene derivatives due to unfavorable interactions with the tips of the 1-pentyne molecules in between the molecules of **1**, as both these molecules have a similar “height” on the surface (Figure 9). In that case, backfilling with a shorter alkyne might improve the interaction between **1** and tetracene. We find similar crystallinity and trends for thinner (30 nm) tetracene layers.

**Figure 8:**
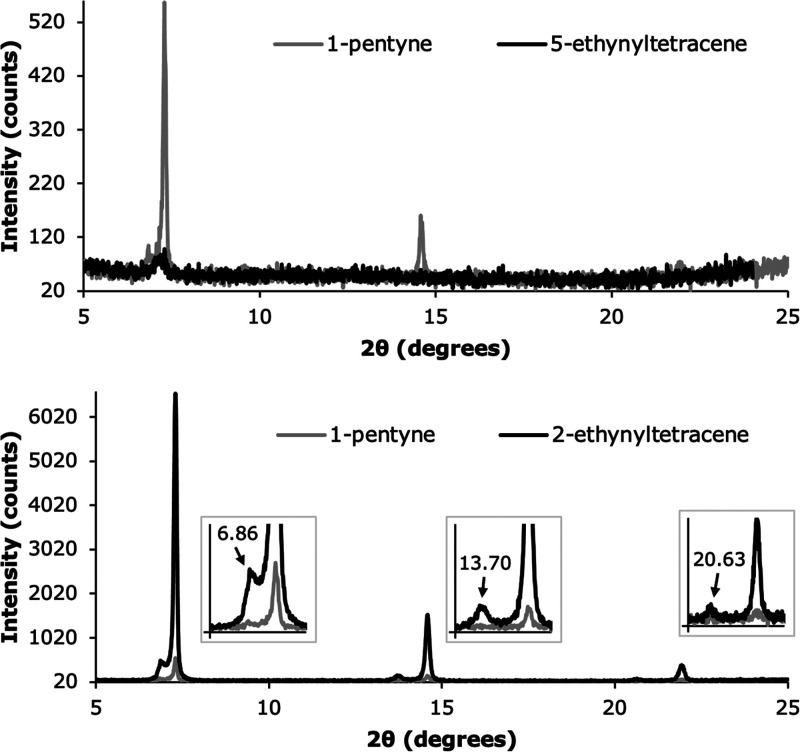
XRD spectra of Si (111) surfaces functionalized with 1 (top) and 2 (bottom), and covered with a 100 nm layer of tetracene. The spectrum of a surface functionalized with 1-pentyne and covered with 100 nm of tetracene is shown in gray as a reference. The spectrum for 2 also contains zoomed-in sections as inserts, to show the smaller peaks belonging to another tetracene polymorph.

**Figure 9:**
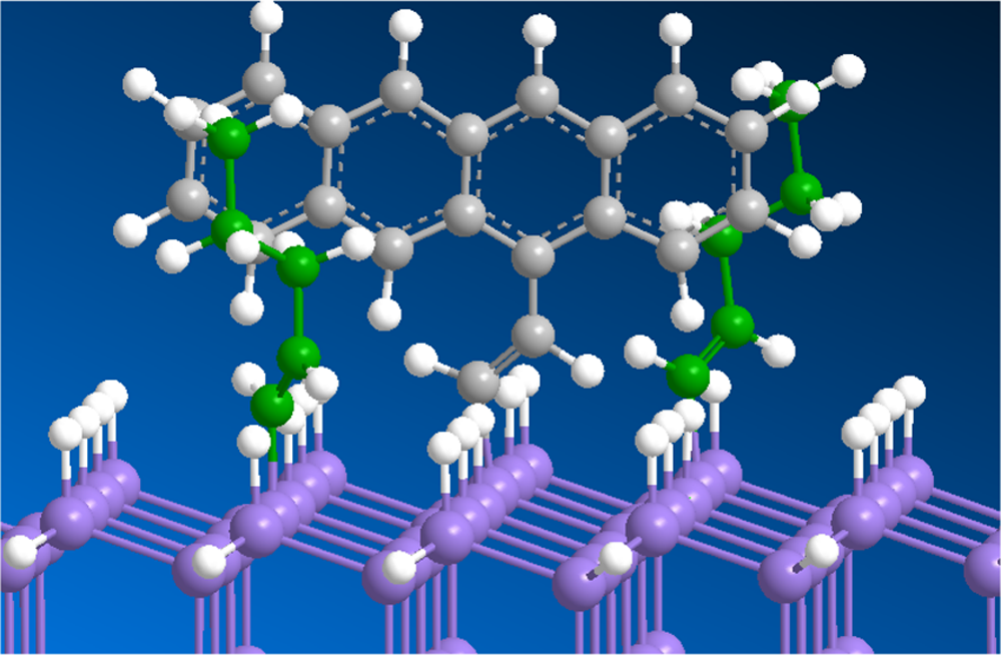
Chemical model for a surface functionalized with 1 and 1-pentyne. For clarity, the 1-pentyne molecules are shown in green.

For surfaces functionalized with **2**, a small orienting effect of the seed layer can be observed in the XRD spectrum (Figure 8, bottom); next to each of the larger peaks also found in the control sample, smaller peaks are found at slightly lower 2θ values, indicating the presence of a different tetracene polymorph in these samples. The 2θ values of these smaller peaks match those of the tetracene polymorph (Tc II) for which faster singlet fission and successful energy transfer to silicon were previously observed.^17,38^ Since neither surfaces functionalized with **1** or 1-pentyne show peaks at these lower 2θ values, this second tetracene polymorph is presumed to be present in the tetracene layers close to the seed layer with the covalently bound molecules **2**. Similar results were also found using 2D XRD measurements (see Supporting Information, Figures S6 and S7). The structural measurements are significant, because they imply that the seed layer induces a change of the crystal structure of the tetracene layer on top. As it has previously been demonstrated that the orientation of tetracene molecules with respect to each other affects the diffusion of the correlated triplet–triplet state and the transfer across the interface,^39^ it would be interesting to further study the singlet fission behavior at the interface between these two crystal structures.

GIWAX measurements were performed to further investigate the degree of order in the tetracene layers on top of the seed layers. Again, surfaces functionalized with **1**, due to their lack of crystallinity in the tetracene layer, showed no signs of ordering in the tetracene layer (see Supporting Information, Figure S7), while surfaces with only 1-pentyne showed a slightly larger peak, indicating a small increase in ordering of the tetracene. However, the sample functionalized with **2** showed excellent ordering, with a large peak at 270°, and two smaller side peaks at a 15° distance (255° and 285°, Figure 10). These results further indicate that **2**, when applied as a covalently bound seed layer, can help orient subsequently deposited tetracene layers into a well-ordered crystal structure.

**Figure 10:**
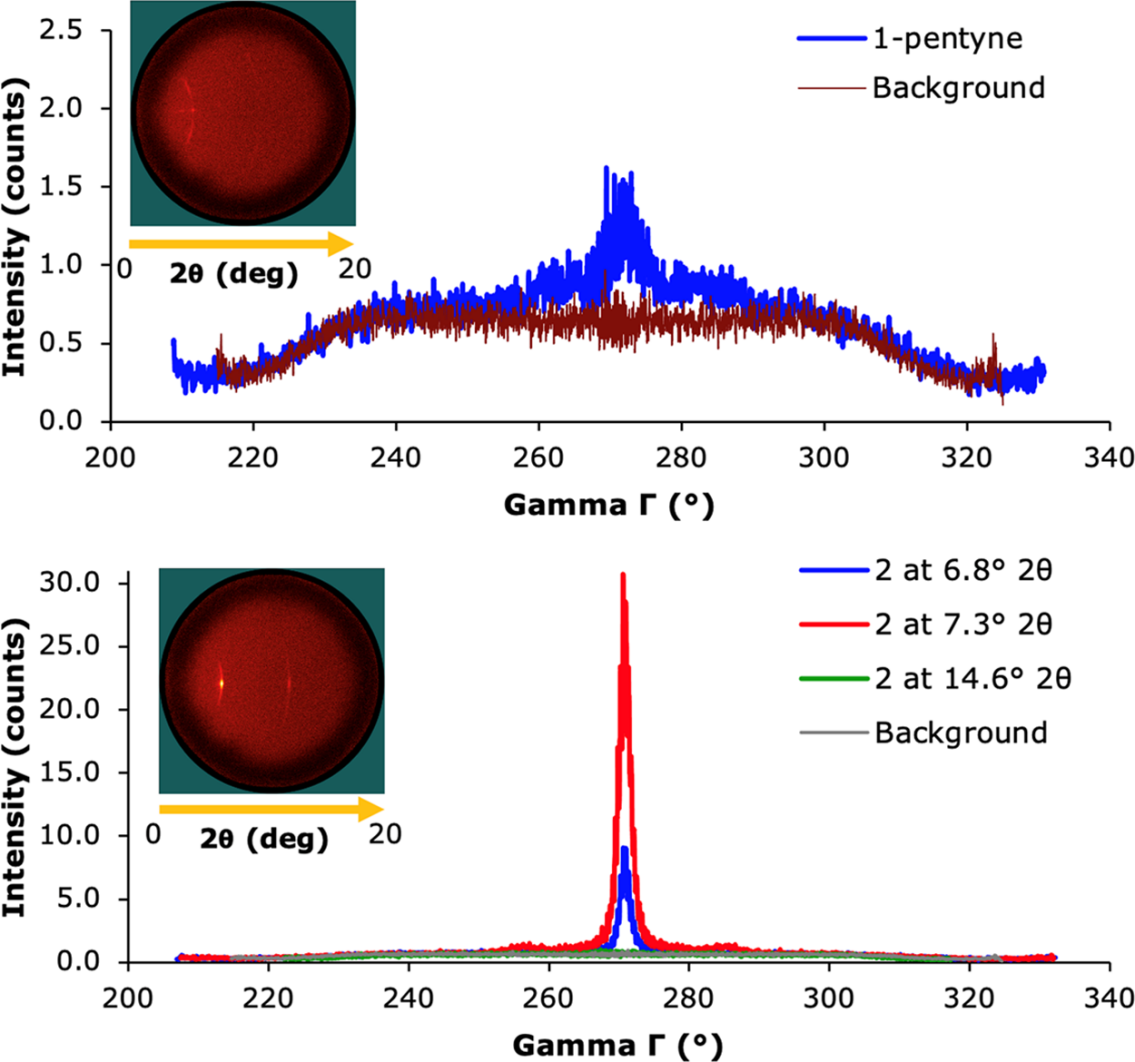
GIWAX spectrum of surfaces functionalized with 1-pentyne (top) and 2 + 1-pentyne (bottom). The spectrum of 1-pentyne was recorded at 7.3°. Background spectra were recorded at 2θ angles where no signal was visible. The inserts show pictures of the surfaces during the GIWAX measurements, where a lighter color indicates a higher-intensity signal.

We next studied the effect of the covalent layer on the triplet exciton energy transfer efficiency. As previous studies indicated that the diffusion length of excitons is greater in a well-ordered, crystalline sample,^40^ we expected a similar superior performance of samples functionalized with **2** in these final measurements, especially when also considering the presence of the tetracene polymorph Tc II near the seed layer, which, as mentioned, has already been shown to transfer more energy to silicon.^17^ To study the triplet energy transfer, we perform magnetic-field dependent photoluminescence measurements on the functionalized silicon surfaces. The magnetic field dependence of the silicon photoluminescence indicates triplet energy transfer if the photoluminescence decreases at high magnetic fields after a small increase at low fields, as described elsewhere.^17^ We deposit 30 nm of tetracene on all three silicon surfaces. However, and to our dismay, no triplet transfer into silicon was found. The magnetic-field dependence follows the opposite trend, with a decrease of photoluminescence at low field and an increase at high field. All three surfaces showed no signs of triplet energy transfer to the silicon layer (Figure 11). The magnetic-field-dependent photoluminescence that is observed instead comes from either direct singlet energy transfer from the tetracene to the silicon, or from indirect transfer after radiative decay of the singlet excitons of the tetracene into photons that are then absorbed by the silicon. Perhaps, as the orientation of the majority of the tetracene layer on the **2**-functionalized surface is still mainly in the less transfer-efficient polymorph Tc I, the small layer of tetracene in the TC II polymorph orientation close to the silicon surface, as observed in the XRD spectra, is not enough to increase the overall energy transfer efficiency. The small differences in the overall magnitude of the PL change could come from different packing of tetracene, which could affect the singlet fission efficiency or PL yield, differences in the surface passivation, or from sample-to-sample variations. We note that we cannot exclude a small amount of dissociation and subsequent charge transfer of triplet excitons at the tetracene/silicon interface.

**Figure 11:**
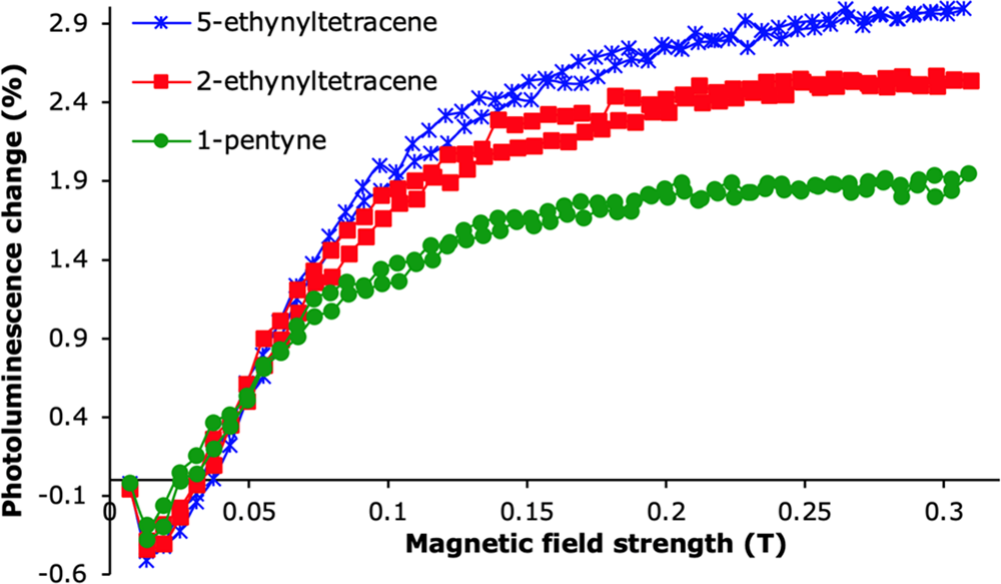
Magnetic-field-dependent silicon photoluminescence measurements of surfaces functionalized with 5-ethynyltetracene (**1**), 2-ethynyltetracene (**2**), and 1-pentyne.

In conclusion, we have shown that it is possible to attach a covalent seed layer of novel tetracene derivatives onto a hydrogen-terminated silicon surface. Modifying the tetracene on the 2-position is in this respect preferred, as this leads to a better preservation of the optical properties compared to the unmodified tetracene. Apart from this, surfaces functionalized with a tetracene moiety bound *via* the 2-position show a greater ability to orient subsequently deposited tetracene layers into the more favorable tetracene polymorph Tc II, and are slightly more resistant against oxidation after backfilling. Unfortunately, there is no (clear) evidence of triplet energy transfer efficiency from tetracene to silicon with any of the functionalized surfaces tested here. In addition, even when a seed layer seems capable of orienting subsequent tetracene layers into a more favorable orientation for energy transfer, this effect seems to be limited to the tetracene layers closest to the surface, with layers further away assuming the preferred, more parallel, orientation of tetracene. This was demonstrated using XRD, where peaks at the 2θ values belonging to the TC II polymorph were observed only after functionalization of the surface with **2**, and only as small peaks next to larger peaks belonging to the Tc I polymorph, indicating the majority of the deposited tetracene layer is still present as the Tc I polymorph. While previous research indicated that the TC II polymorph and good crystallinity close to the surface is required for energy transfer, our results show that this is not sufficient. Further research into the effect of stacking of the organic crystals close to the interface, for example, when seed layers with longer linker groups and alternative linker positions are used, together with the aforementioned need to optimize the backfilling process, is therefore needed to make singlet fission-sensitized silicon solar cells a viable alternative to conventional solar cells.

## Supporting Information Available

Additional experimental details, materials, and methods, including synthesis of the tetracene derivatives, supplementary spectroscopy, and XRD and GIWAX measurements (PDF)
