# Correction to “Toward Improving Triplet Energy Transfer from Tetracene to Silicon Using a Covalently Bound Tetracene Seed Layer”

**DOI:** 10.1021/acs.jpclett.3c01487

**Published:** 2023-06-01

**Authors:** Alyssa F. J. van den Boom, Silvia Ferro, María C. Gelvez-Rueda, Han Zuilhof, Bruno Ehrler

In the original version, only Bruno Ehrler is listed as a corresponding author, but both Han Zuilhof and Bruno Ehrler are corresponding authors.

