# Supplementary material for: Toward Improving Triplet Energy Transfer from Tetracene to Silicon Using a Covalently Bound Tetracene Seed Layer
Source: J Phys Chem Lett. 2023 May 8;14(19):4454–61. doi: 10.1021/acs.jpclett.3c00589 (PMC10201576; doi:10.1021/acs.jpclett.3c00589)
Supplement: Supplementary file 1 — jz3c00589_si_001.pdf [file jz3c00589_si_001.pdf]

Supporting Information for

Towards improving triplet energy transfer from tetracene to silicon using a covalently-bound tetracene seed layer

Alyssa F. J. van den Boom[1], Silvia Ferro[2], María Gelvez Rueda[2], Han Zuilhof[1,3*] and Bruno Ehrler[2*]

[1] Laboratory of Organic Chemistry, Wageningen University, 6708 WE Wageningen, The Netherlands;
[2] Center for Nanophotonics, AMOLF, Science Park 104, 1098 XG Amsterdam, The Netherlands
[3] School of Pharmaceutical Science and Technology, Tianjin University, Tianjin 300072, China;

General information

All chemicals were purchased from either Merck Life Science N.V. or abcr, and used as received. Double side polished silicon(111) wafers with a diameter of 76.2±0.3 mm were purchased from SIEGERT WAFER GmbH (FZ growth, prime grade, N type, Ph-doped, 381±25 µm thickness), and cut into 1x1 cm squares using a laser cutter.

NMR measurements were conducted on a 400 MHz Bruker Avance III at 298K, and the resulting data were analyzed using MestReNova software, version 14.1.0-24037. Spectra were calibrated relative to signals corresponding to the non-deuterated solvents ($CDCl_3$ solvent peak) – at 7.26 ppm for ^1H spectra, and 77.16 ppm for ^{13}C spectra. For spectra recorded in DMSO-d6, ^1H spectra were calibrated to the non-deuterated solvent peak at 2.50 ppm. Abbreviations used in the description of NMR data are as follows: chemical shift (δ = ppm), multiplicity (s = singlet, d = doublet, t = triplet, q = quartet, p= pentet, sextet = sext, h = heptet, dd = double boublet, dt = doublet of triplets, m = multiplet), coupling constant (*J*, Hz).

High-resolution mass spectra (HRMS) were recorded on a Thermo Scientific Exactive 1.1 with an orbitrap mass analyzer, using a DART gun from Ion Sense. The temperature of the DART gun was set to 450-550 °C, with a helium gas flow. Data were analyzed using Thermo Xcalibur software, version 2.2 SP1.48.

IR spectra were recorded on a Bruker Tensor 27 spectrometer equipped with a diamond ATR accessory (64 scans; 4 cm^{-1} resolution; range 4000-350 cm^{-1}). Strong or indicative peaks in the region 4000-1000 cm^{-1} are mentioned for each novel compound.

IR-RAS spectra were recorded on a Bruker Tensor 27 with MCT detector equipped with a Seagull™ Variable Angle Reflection Accessory. A germanium crystal was used to perform Ge-ATR measurements using P polarization and a mirror angle of 68°. A total of 2048 scans were performed per sample, with a resolution of 4 cm^{-1}.

XPS measurements were performed on a Jeol JPS-9200 photoelectron spectrometer, using a 12 kV and 20 mA monochromatic Al Kα source. The analyzer pass energy was 10 eV, and the take-off angle between sample and detector was set at 80°. Data was analyzed using the program CasaXPS, version 2.3.18PR1.0.

Optical properties (in DCM) were studied using an Edinburgh Instruments FLS900 fluorescence spectrometer, using a 450 W xenon lamp as the excitation source. For the UV-Vis absorption spectra, a Varian Cary 50 UV-Vis spectrophotometer was used, with a scan rate of 600 nm/min. Absorption spectra were corrected for solvent absorption.

Tetracene deposition 30 nm or 100 nm tetracene layers were deposited on silicon surfaces using an evaporation chamber by Angstrom Engineering Inc, at a base pressure below 7*10^{-7} mbar. Tetracene was purchased from Sigma-aldrich (99.99% purity) and used as is. The deposition was 1Å/s in all cases.

Magnetic-field-dependent photocurrent measurements were performed using a home-built setup. The magnetic field is applied by an electromagnet, made up by two Helmholtz coils and calibrated using a Hall effect sensor. The magnetic field is applied by sending a current of up to 5 Amperes through the magnet, resulting in a magnetic field of up to 0.35 T. The field is oriented parallel to the sample surface. The excitation source is a 520 nm diode laser, installed in a Thorlabs temperature controlled laser housing. The cw laser power is around 10 mW with a laser spot size of approx. 1 mm. The photocurrent is measured with a Keithly 2636A Source measure unit.

1D XRD measurements were conducted using a Bruker D8 Advance diffractometer (Bragg-Brentano) with a Co-Kα source (λ=1.7889 Å, 35 kV, 40 mA) and a Lynxeye position sensitive detector

2D GIWAX XRD measurements were conducted using a Bruker D8 Discover with a Co-Kα GIWAX source (1mS microfocus tube, 50 kV, 1000 mA) and a VANTEC500 2D-detector. Samples were measured under the following conditions: 2.0 mm collimator, 600 s/step, and a 4° X-ray incidence angle.

Experimental details

Synthesis of tetracene

Tetracene was prepared according to the procedure described in Kulkarni *et al.* (2018).[1] In short, 2.50 g (9.68 mmol, 1 equiv) of 5,12-naphthacenequinone was dissolved in 220 ml each of methanol and THF. While stirring, 1.65 g (43.62 mmol, 4.5 equiv) of $NaBH_4$ was slowly added, and the reaction mixture was allowed to stir for another hour at room temperature. Then, the mixture was neutralized with (glacial) acetic acid, and transferred to an extraction funnel. Here, 150 ml of both brine and DCM are added, and the organic layer is separated. The organic layer is washed with 2x 150 ml of brine, and the combined water layers are back-extracted with 150 ml of DCM. The organic layers are combined, dried over magnesium sulfate, and concentrated on a rotavapor to yield 2.34 g (8.93 mmol) of 5,12-dihydrotetracene-5,12-diol as a yellow solid.

This solid was dissolved in 270 ml of THF, and a mixture of 3.41 g (18.00 mmol, 2 equiv) of $SnCl_2$ in 4.3 ml of 37% HCl was added dropwise under vigorous stirring. After addition was complete, the reaction was allowed to stir for 1 hour at room temperature. Then, the mixture was filtered, and the solid washed with 1M HCl, water, and methanol, to give 1.32 g (5.78 mmol) of tetracene as orange needles.

^{1}H NMR (400 MHz, $CDCl_3$): δ 8.67 (s, 4H), 8.00 (dd, *J* = 6.2, 3.3 Hz, 4H), 7.40 (dd, *J* = 6.8, 3.2 Hz, 4H). These values match those reported by Kulkarni *et al.* (2018).

Synthesis of 5-ethynyltetracene (1)

5-ethynyltetracene was prepared according to the procedure described by Korovina *et al.* (2016).[2]

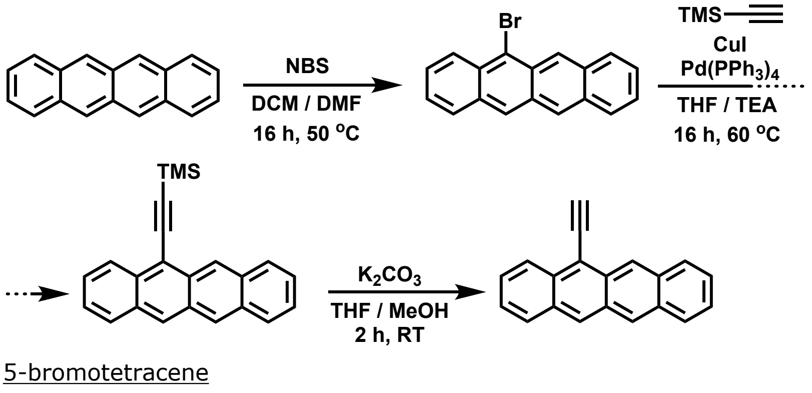

5-bromotetracene

In a three-necked flask equipped with a stir bar, 1.00 g (4.38 mmol, 1 equiv) of tetracene was suspended in 250 ml of dry DCM, and the suspension was sonicated for 30 minutes. Meanwhile, an addition funnel

was charged with 0.86 g (4.83 mmol, 1.1 equiv) of n-bromosuccinimide (NBS), 70 ml of dry DCM, and 15 ml of dry DMF. This solution was flushed with argon for 10 minutes, after which the addition funnel was installed onto the three-necked flask under argon flow. A condenser was added, and the tetracene suspension was heated to 50 °C under argon. Then, the NBS solution was added dropwise, over a period of 2h. Next, the reaction mixture was allowed to stir overnight at 50 °C, in the dark, under argon. The mixture was cooled down to room temperature, and the DCM was removed on a rotavapor. 30 ml of methanol was added to the resulting slurry, and the mixture was cooled down to 0 °C. 5 ml of water was added dropwise, and the red solids were collected by vacuum filtration. The solids were washed with cold water and methanol to yield 1.25 g (4.08 mmol, 93%) of 5-bromotetracene as a red solid.
^{1}H NMR (400 MHz, $CDCl_3$): δ 9.14 (s, 1H), 8.64 (d, *J* = 3.8 Hz, 2H), 8.48 (d, *J* = 8.9 Hz, 1H), 8.08 (d, *J* = 7.4 Hz, 1H), 8.02 – 7.94 (m, 2H), 7.56 – 7.50 (m, 1H), 7.48 – 7.38 (m, 3H).

5-((trimethylsilyl)ethynyl)tetracene:
The 5-bromotetracene was transferred into a three-necked flask, together with 34 mg (0.18 mmol) of CuI, 162 mg (0.14 mmol) of $Pd(PPh_3)_4$, and 30 ml of dry THF. The resulting solution was flushed with argon for 15 min. Meanwhile, an addition funnel was filled with a solution of 0.90 ml (6.37 mmol, 1.6 equiv) of (trimethylsilyl)acetylene in 25 ml of triethylamine, and this solution was also flushed with argon for 15 min, before the addition funnel was installed onto the three-necked flask. A condenser was added, and the 5-bromotetracene solution was heated to 60 °C. The (trimethylsilyl)acetylene solution was added dropwise, and the reaction mixture was allowed to stir overnight at 60 °C, in the dark, under argon atmosphere.
After cooling down, the reaction mixture was added to a separation funnel containing 50 ml of DCM. The organic layer was washed with 3x 20 ml of a saturated ammonium chloride solution, and the combined water layers were back-extracted with 20 ml of DCM. The organic layers were combined, dried over $MgSO_4$, and concentrated on a rotavapor. The crude product was then further purified using flash chromatography (silica gel, mobile phase: hexane) to yield 1.01 g (3.10 mmol, 76%) of 5-((trimethylsilyl)ethynyl)tetracene as a red solid.
^{1}H NMR (400 MHz, $CDCl_3$): δ 9.18 (s, 1H), 8.66 (s, 2H), 8.55 (dd, *J* = 8.8, 1.0 Hz, 1H), 8.10 – 8.07 (m, 1H), 8.03 – 7.97 (m, 2H), 7.55 – 7.50 (m, 1H), 7.46 – 7.41 (m, 3H), 0.48 (s, 9H). These values match those reported in [2].

5-ethynyltetracene:
0.15 g (0.46 mmol, 1 equiv) of 5-((trimethylsilyl)ethynyl)tetracene was added to a round bottom flask under argon, along with 0.23 g (1.66 mmol, 3.6 equiv) of potassium carbonate. Next, still under argon, 4.5 ml of dry THF, and 2.8 ml of dry methanol were added. The resulting suspension was allowed to stir for 2h, in the dark, under argon. Afterwards, the solvent was removed on a rotavapor, and the product was passed through a silica plug using dry DCM. The solvent was again evaporated, and the product was transported into a glovebox, where it was immediately used for surface functionalisation.
^{1}H NMR (400 MHz, $CDCl_3$): δ 9.26 (s, 1H), 8.71 (d, *J* = 6.2 Hz, 2H), 8.63 (d, *J* = 7.8 Hz, 1H), 8.16 – 8.10 (m, 1H), 8.08 – 8.01 (m, 2H), 7.61 – 7.54 (m, 1H), 7.53 – 7.45 (m, 3H), 4.18 (s, 1H). These values match those reported in [2].

Synthesis of 2-ethynyltetracene (2)
For the synthesis of 2-ethynyltetracene, 2-bromotetracene was first prepared according to a procedure from Zhao *et al.* (2020).[3] Then, an adapted procedure from the one described above for 5-ethynyltetracene was followed.

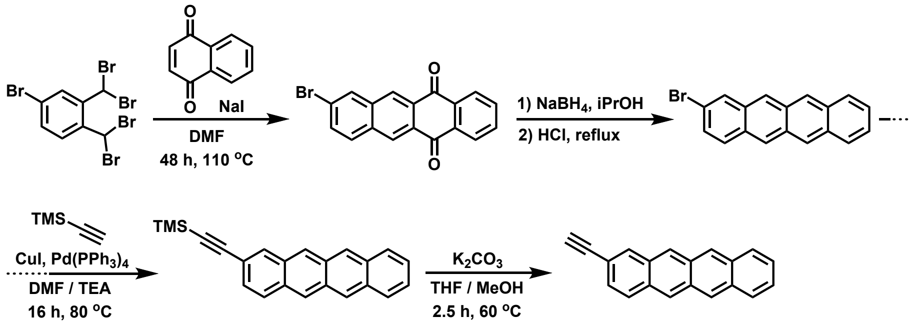

8-bromotetracene-5,12-dione

A round bottom flask under argon was charged with 3.94 g (26.28 mmol, 4.2 equiv) of NaI, and 1.00 g (6.31 mmol, 1 equiv) of 1,4-naphthoquinone. 30 ml of DMF was added, followed by 3.29 g (6.57 mmol, 1.04 equiv) of α,α,α,α-tetrabromo-4-bromo-*o*-xylene. The resulting mixture was stirred at 110 °C for 48h. After cooling down, the precipitated solid was collected using vacuum filtration, washed with water, methanol, and acetone, and dried under vacuum to yield 1.41 g (4.17 mmol, 66%) of 8-bromotetracene-5,12-dione as a golden-yellow, shiny solid.

^{1}H NMR (400 MHz, $CDCl_3$): δ 8.84 (s, 1H), 8.78 (s, 1H), 8.41 (dd, *J* = 5.8, 3.4 Hz, 2H), 8.28 (s, 1H), 7.99 (d, *J* = 8.8 Hz, 1H), 7.85 (dd, *J* = 6.0, 3.3 Hz, 2H), 7.78 (d, *J* = 8.7 Hz, 1H). These values match data from [3].

2-bromotetracene

The fresh 8-bromotetracene-5,12-dione was dissolved in 140 ml of isopropanol, and 2.18 g (57.63 mmol, 14 equiv) of $NaBH_4$ was added. A condenser was installed onto the flask, and the reaction mixture was allowed to reflux at 95 °C for 24h in the dark. After cooling down, the mixture was placed on an ice bath, and 140 ml of a 2M aqueous HCl solution was added dropwise. The mixture was heated to 105 °C, and allowed to reflux for 3h. After cooling, the solids were collected using vacuum filtration, and washed with water, methanol, acetone, and hexane, to yield 0.97 g (3.14 mmol, 75%) of 2-bromotetracene as an orange solid.

Note: *the solubility of 2-bromotetracene is exceptionally low in common organic solvents.*

^{1}H NMR (400 MHz, $CDCl_3$): δ 8.71 – 8.61 (m, 3H), 8.57 (s, 1H), 8.18 (s, 1H), 8.03 – 7.97 (m, 2H), 7.88 (d, *J* = 9.1 Hz, 1H), 7.46 – 7.37 (m, 3H). These values match data from [3].

2-((trimethylsilyl)ethynyl)tetracene

The thus prepared 2-bromotetracene was dissolved in 28 ml of dry DMF in a three-necked flask under argon, and 32 mg (0.17 mmol) CuI and 152 mg (0.13 mmol) $Pd(PPh_3)_4$ were added. The resulting suspension was flushed with argon for 15 minutes. Meanwhile, 0.7 ml (5.16 mmol, 1.6 equiv) of (trimethylsilyl)acetylene was dissolved in 24 ml of triethylamine in an addition funnel under argon. The addition funnel was installed onto the three-necked flask, along with a condenser, and the flask was heated to 85 °C. The (trimethylsilyl)acetylene solution was added dropwise, and the reaction was allowed to stir overnight at 85 °C, in the dark, under argon.

The mixture was cooled down, diluted with hexane, and cooled down further on an ice bath. The solids were collected using vacuum filtration, and washed with water, methanol, acetone, and hexane, to yield 0.67 g (2.06 mmol, 66%) of 2-((trimethylsilyl)ethynyl)tetracene as a red solid.

Note: *the solubility of 2-((trimethylsilyl)ethynyltetracene is exceptionally low in common organic solvents.*

^{1}H NMR (400 MHz, $CDCl_3$): δ 8.65 (d, *J* = 6.7 Hz, 2H), 8.60 (s, 2H), 8.17 (s, 1H), 8.00 (dd, *J* = 6.6, 3.2 Hz, 2H), 7.91 (d, *J* = 8.9 Hz, 1H), 7.41 (dd, *J* = 6.6, 3.2 Hz, 2H), 7.36 (dd, *J* = 8.8, 1.5 Hz, 1H), 0.31 (s, 9H). **^{13}C NMR (101 MHz, $CDCl_3$):** δ 132.9, 128.4, 127.4, 126.8, 126.5, 125.6, 0.2. **IR:** $\bar{\upsilon}_{max}$ = 2955, 2899, 2146, 1462, 1294, 1248, 1169, 955, 904, 868, 837, 802, 758, 733, 642, 467 cm^{-1} **HRMS:** *m/z* $C_{23}H_{21}Si^+$ ($[M+H]^+$): calculated 325.1407, found 325.1406.

2-ethynyltetracene

To deprotect the 2-((trimethylsilyl)ethynyl)tetracene for use in surface functionalisation, 25 mg (0.077 mmol) of the compound was added to a Schlenk flask equipped with a stir bar, along with 38 mg (0.275 mmol, 3.6 equiv) of K_2CO_3. The flask was flushed with argon, after which 4.0 ml of THF and 2.7 ml of methanol were added under argon flow. The flask was sealed, heated to 60 °C under argon, and stirred in the dark for 2.5 h. Afterwards, the mixture was cooled down to room temperature, and the solvents were evaporated. The crude product was passed through a silica plug with ~500 ml of hot, dry DCM, and the solvents were again evaporated. The thus purified product was transferred into a glovebox, where it was immediately used for surface functionalisation.

Note: *the solubility of 2-ethynyltetracene is exceptionally low in common organic solvents.*

^{1}H NMR (400 MHz, $CDCl_3$): δ 8.66 (d, *J* = 5.4 Hz, 2H), 8.63 (s, 2H), 8.21 (s, 1H), 8.00 (dd, *J* = 6.5, 3.3 Hz, 2H), 7.94 (d, *J* = 8.9 Hz, 1H), 7.42 (dd, *J* = 6.7, 3.2 Hz, 2H), 7.38 (dd, *J* = 8.8, 1.6 Hz, 1H), 3.22 (s, 1H). This compound is unstable, and could therefore not be further characterized. Full characterization was therefore performed on 2-((trimethylsilyl)ethynyl)tetracene.

Synthesis of 2-fluoro-6-((trimethylsilyl)ethynyl)tetracene (1F)

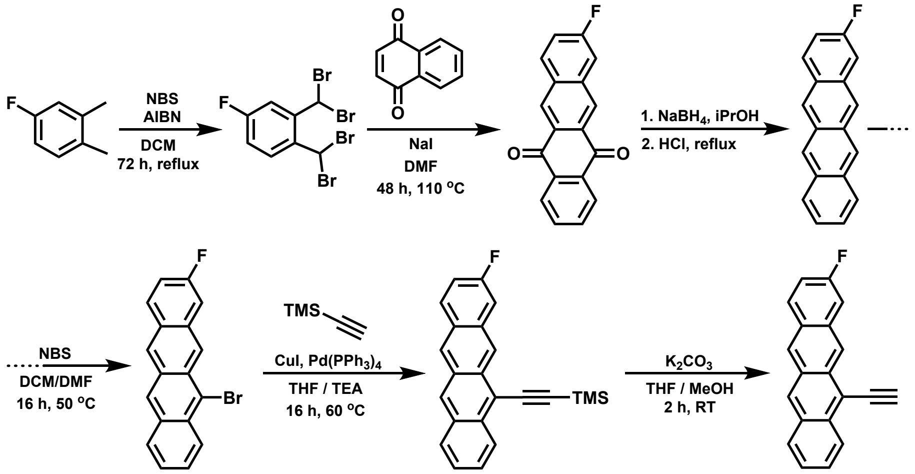

4-fluoro-α,α,α,α-tetrabromoxylene was prepared according to the procedure described by Zhao *et al.* (2020).[3] 7.10 g (40.21 mmol) of n-bromosuccinimide (NBS) was suspended in 38 ml of DCM, and to this were added 2.5 g (20.14 mmol) of 4-fluoro-*o*-xylol and 0.11 g (0.65 mmol) of azobis(isobutyronitrile) (AIBN). This mixture was then refluxed for 72 h, adding another 7.10 g of NBS and 0.11 g of AIBN after 24 h and 48 h. After cooling down, the mixture was filtered, and the solids washed with DCM. The combined filtrate and washing solution were then washed 3x with water, aqueous $Na_2S_2O_3$, and brine. The organic layer was isolated, dried over $MgSO_4$, and concentrated on a rotavap. The crude product was further purified using column chromatography (silica gel, eluent: hexane) to yield 3.91 g (8.88 mmol, 44%) of off-white solids.

8-fluoro-5,12-naphthacenequinone was prepared in the same way as 8-bromotetracene-5,12-dione – starting with 3.69 g (8.39 mmol) of 4-fluoro-α,α,α,α-tetrabromoxylene – to yield 0.68 g (2.46 mmol, 29%) of gold-yellow solids.
^{1}H NMR (400 MHz, $CDCl_3$): δ 8.87 (s, 1H), 8.80 (s, 1H), 8.41 (dd, *J* = 6.1, 3.1 Hz, 2H), 8.13 (dd, *J* = 9.0, 5.5 Hz, 1H), 7.84 (dd, *J* = 5.8, 3.3 Hz, 2H), 7.73 (dd, *J* = 9.2, 2.5 Hz, 1H), 7.49 (td, *J* = 8.7, 2.5 Hz, 1H). **^{13}C NMR (101 MHz, $CDCl_3$):** δ 183.07, 182.85, 164.04, 161.51, 134.50, 134.43, 133.03, 132.94, 129.70, 128.84, 128.78, 127.73, 127.71, 120.41, 120.15, 113.55, 113.35.

2-fluototetracene was prepared from 8-fluoro-5,12-naphthacenequinone according to the procedure described for 2-bromotetracene, to yield 0.52 g (2.11 mmol, 86%) of orange solids.
***Note:** the solubility of 2-fluorotetracene is exceptionally low in common organic solvents.*
^{1}H NMR (400 MHz, $CDCl_3$): δ 8.67 (d, *J* = 6.9 Hz, 2H), 8.61 (d, *J* = 17.0 Hz, 2H), 8.05 – 7.95 (m, 3H), 7.56 (d, *J* = 10.2 Hz, 1H), 7.44 – 7.38 (m, 2H), 7.22 (d, *J* = 9.5 Hz, 1H).
These values match previously reported data for this compound.[4]

11-bromo-2-fluorotetracene was prepared from 0.43 g (1.75 mmol) of 2-fluorotetracene according to the procedure described for 5-bromotetracene, to yield 0.52 g (1.61 mmol, 92%) of orange solids.
^{1}H NMR (400 MHz, $CDCl_3$): δ 9.08 (d, *J* = 8.5 Hz, 1H), 8.67 (s, 2H), 8.49 (d, *J* = 9.0 Hz, 1H), 8.09 (d, *J* = 10.5 Hz, 1H), 8.04 – 7.96 (m, 2H), 7.61 – 7.39 (m, 3H).

5-((trimethylsilyl)ethynyl)-8-fluorotetracene was prepared from 0.52 g of 11-bromo-2-fluorotetracene according to the procedure described for 5-((trimethylsilyl)ethynyl)tetracene, to yield 0.55 g (1.61 mmol, 100%) of red solids.
^{1}H NMR (400 MHz, $CDCl_3$): δ 9.09 (d, *J* = 13.4 Hz, 1H), 8.65 – 8.60 (m, 2H), 8.12 – 8.05 (m, 1H), 8.01 – 7.96 (m, 2H), 7.57 – 7.50 (m, 1H), 7.50 – 7.40 (m, 2H), 7.23 (ddd, *J* = 7.8, 2.5, 1.5 Hz, 1H), 0.49 (d, *J* = 1.2 Hz, 9H). **^{13}C NMR (101 MHz, $CDCl_3$):** δ 131.6, 131.5, 128.5, 128.4, 128.3 (d, *J* = 2.9 Hz), 128.1, 127.8, 127.4, 127.1, 126.7, 125.7, 125.3, 125.1, 124.7, 124.2 (d, *J* = 8.5 Hz), 117.6, 109.5 (d, *J* = 20.7 Hz), 108.5 (d, *J* = 22.6 Hz), 101.5 (d, *J* = 23.1 Hz). **^{19}F NMR (376 MHz, $CDCl_3$):** δ -111.13 (m). **IR:** $\tilde{\upsilon}_{max}$

= 3051, 2958, 2139, 1637, 1468, 1248, 1174, 1157, 891, 839, 733, 644, 463, 436. **HRMS:** *m/z* $C_{23}H_{20}FSi^+$ ($[M+H]^+$): calculated 343.1313, found 343.1311.

11-ethynyl-2-fluorotetracene was prepared from 0.2 g (0.58 mmol) of 5-((trimethylsilyl)ethynyl)-8-fluorotetracene according to the procedure for 5-ethynyltetracene. Quantitative yield.
^{1}H NMR (400 MHz, $CDCl_3$): δ 9.14 (d, *J* = 12.8 Hz, 1H), 8.68 (t, *J* = 4.7 Hz, 2H), 8.57 (d, *J* = 9.4 Hz, 1H), 8.08 (d, *J* = 7.1 Hz, 1H), 8.04 – 7.99 (m, 2H), 7.60 – 7.50 (m, 1H), 7.50 – 7.41 (m, 2H), 7.24 (s, 1H), 4.15 (s, 1H). This compound is unstable, and could therefore not be characterized further. Full characterization was therefore performed on 5-((trimethylsilyl)ethynyl)-8-fluorotetracene.

Synthesis of 2-ethynyl-6,11-bis(trifluoromethyl)tetracene (2F)
For the synthesis up to 2-bromo-6,11-bis(trifluoromethyl)tetracene, an adapted procedure from Schwaben *et al.* (2015) was used.[5] All following products were prepared using protocols already described above.

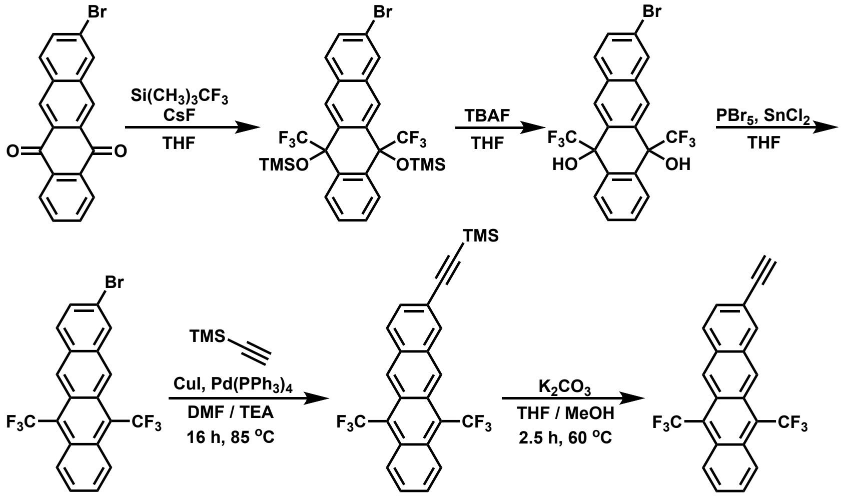

2-bromo-6,11-bis(trifluoromethyl)tetracene
A round bottom flask under argon was charged with 1.32 g of 8-bromotetracene-5,12-dione (3.91 mmol, 1 equiv) and 26 ml of dry THF. To this, 1.28 ml (8.60 mmol, 2.2 equiv) of trimethyl(trifluoromethyl)silane was added, and the suspension was cooled to 0 °C. 5.9 mg (0.04 mmol, 1 mol%) of CsF was added, and the reaction was stirred for 10 minutes. Then, the mixture was allowed to warm to room temperature, and stirred for another 1.5 h. The solvent was evaporated, and the crude product was purified using column chromatography (silica gel, 15:2 hexane/MTBE) to yield the intermediate ((8-bromo-5,12-bis(trifluoromethyl)-5,12-dihydrotetracene-5,12-diyl)bis(oxy))bis(trimethylsilane). **^{1}H NMR (400 MHz, $CDCl_3$):** δ 8.44 (d, *J* = 2.4 Hz, 1H), 8.39 (d, *J* = 2.5 Hz, 1H), 8.16 (s, 1H), 8.01 (dt, *J* = 5.9, 2.9 Hz, 2H), 7.85 (d, *J* = 8.7 Hz, 1H), 7.67 (dd, *J* = 8.8, 2.0 Hz, 1H), 7.55 (dd, *J* = 6.1, 3.4 Hz, 2H), -0.05 (d, *J* = 4.4 Hz, 18H).
This intermediate was dissolved in 27 ml of dry THF, and the solution was cooled down to 0 °C. Then, 8.6 ml of a 1M solution of TBAF in dry THF (8.60 mmol, 2.2 equiv) was added dropwise, after which the reaction mixture was allowed to warm to room temperature. The reaction was stirred for 30 minutes, and transferred to an extraction funnel. DCM and brine (27 ml each) were added, and the layers were separated. The water phase was extracted with 3x 27 ml of DCM. The organic layers were combined, dried, and concentrated under vacuum. The crude product was purified using column chromatography (silica gel, 5:1 to 1:1 hexane/MTBE) to yield the intermediate 8-bromo-5,12-bis(trfluoromethyl)-5,12-dihydrotetracene-5,12-diol. **^{1}H NMR (400 MHz, DMSO-d6):** δ 8.57 (d, *J* = 5.0 Hz, 2H), 8.46 (d, *J* = 2.0 Hz, 1H), 8.11 (d, *J* = 8.8 Hz, 1H), 8.02 (dd, *J* = 5.8, 3.3 Hz, 2H), 7.81 (d, *J* = 7.5 Hz, 2H), 7.74 (dd, *J* = 8.8, 2.0 Hz, 1H), 7.61 (dt, *J* = 7.2, 3.6 Hz, 2H).
This intermediate was again dissolved in 22 ml of dry THF at room temperature, and 6.7 g (14.82 mmol, 4 equiv) of phosphorus pentabromide was added in portions. The reaction mixture was heated to 50 °C, and stirred for 18 h. After this time, 7.01 g (37 mmol, 10 equiv) of $SnCl_2$ was added, and the reaction was stirred for another 10 minutes at 50 °C. Next, the mixture was cooled to room temperature, and diluted with 215 ml of diethyl ether in an extraction funnel. 110 ml of a saturated solution of $NaHCO_3$ was added

slowly, and the organic phase was isolated, washed with 45 ml of brine, dried, and concentrated under vacuum. The resulting solids were washed with hexane, and dried to yield 0.94 g (2.11 mmol, 54%) of 2-bromo-6,11-bis(trifluoromethyl)tetracene. **^{1}H NMR (400 MHz, $CDCl_3$):** δ 8.52 (d, *J* = 2.2 Hz, 1H), 8.46 (d, *J* = 2.2 Hz, 1H), 8.13 (d, *J* = 1.9 Hz, 1H), 8.09 (dt, *J* = 5.8, 2.8 Hz, 2H), 7.83 (d, *J* = 8.8 Hz, 1H), 7.65 (dd, *J* = 8.7, 1.9 Hz, 1H), 7.59 (dd, *J* = 6.0, 3.4 Hz, 2H).

<u>((6,11-bis(trifluoromethyl)tetracene-2-yl)ethynyl)trimethylsilane</u>
((6,11-bis(trifluoromethyl)tetracene-2-yl)ethynyl)trimethylsilane was prepared in the same way as 2-((trimethylsilyl)ethynyl)tetracene, but using column chromatography (silica gel, 30% diethyl ether in hexane) to purify the final product as beige solids. **^{1}H NMR (400 MHz, $CDCl_3$):** δ 8.49 (dd, *J* = 7.7, 2.2 Hz, 2H), 8.14 – 8.05 (m, 3H), 7.89 (d, *J* = 8.5 Hz, 1H), 7.65 – 7.57 (m, 3H), 0.30 (s, 9H). **^{13}C NMR (101 MHz, $CDCl_3$):** δ 133.23, 132.76, 132.58, 132.34, 130.84, 130.79, 130.57, 130.07, 128.82 (q, *J* = 2.7 Hz), 128.64 (q, *J* = 3.0 Hz), 128.41, 125.64, 122.72, 104.81, 96.41, 73.63 (q, *J* = 28.0 Hz), 73.61 (q, *J* = 28.0 Hz), 0.08. **^{19}F NMR (376 MHz, $CDCl_3$):** δ -78.28 (s), -78.39 (s). **IR:** $\tilde{\upsilon}_{max}$ = 3078, 2958, 2152, 1331, 1252, 1205, 1159, 1034, 976, 912, 847, 760, 737, 688, 640. **HRMS:** *m/z* $C_{25}H_{19}F_6Si^+$ ($[M+H]^+$): calculated 461.1155, found 461.1192.

After deprotection using the same protocol as for 2-ethynyltetracene, the title compound 2-ethynyl-6,11-bis(trifluoromethyl)tetracene was obtained as an off-white solid in quantitative yield. This compound is unstable, and could therefore not be characterized. Full characterization was therefore performed on ((6,11-bis(trifluoromethyl)tetracene-2-yl)ethynyl)trimethylsilane.

Surface functionalization and backfilling
Double side polished silicon (111) surfaces of 1x1 cm were cleaned before etching and functionalisation by sonicating them for 10 minutes each in isopropanol, hexane, acetone, and DCM. After the final sonication step, the surfaces were blown dry with a stream of nitrogen, and submitted to a 10-minute plasma treatment in a plasma oven, using oxygen gas to generate the plasma. Then, the surfaces were transported into an oxygen-free glovebox (O_2 = 0%). Here, they were etched with a solution of 40% NH_4F in water for 15 minutes, under constant bubbling with nitrogen gas to remove bubbles formed during etching. After etching, the surfaces were washed extensively with deoxygenated water, and blown dry with a stream of nitrogen. The thus prepared surfaces were immediately used for functionalisation by fully immersing them in either an argon-purged solution of tetracene derivative in dry toluene, or neat 1-pentyne for the reference surface. The immersed surfaces were heated to 90 °C, and allowed to react overnight. The next day, the surfaces were removed from the solution, and sonicated for 10 minutes in dry DCM to remove any physisorbed 1-pentyne or tetracene derivative. The surfaces functionalised with tetracene derivative were then submerged in neat 1-pentyne for backfilling. Again, the surfaces were allowed to react at 90 °C overnight, and cleaned by sonicating in dry DCM.

Supplementary figures

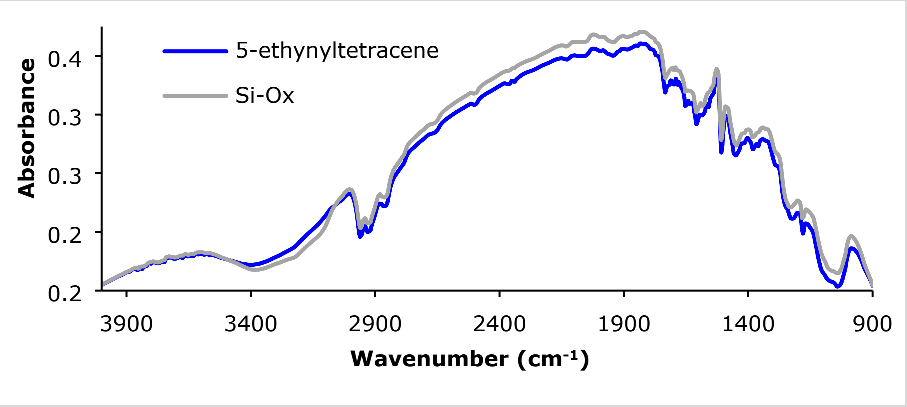

Figure S1: IR-RAS spectrum of a Si(111) surface functionalized with **1**. The spectrum of a cleaned, non-etched Si(111) is shown as a background.

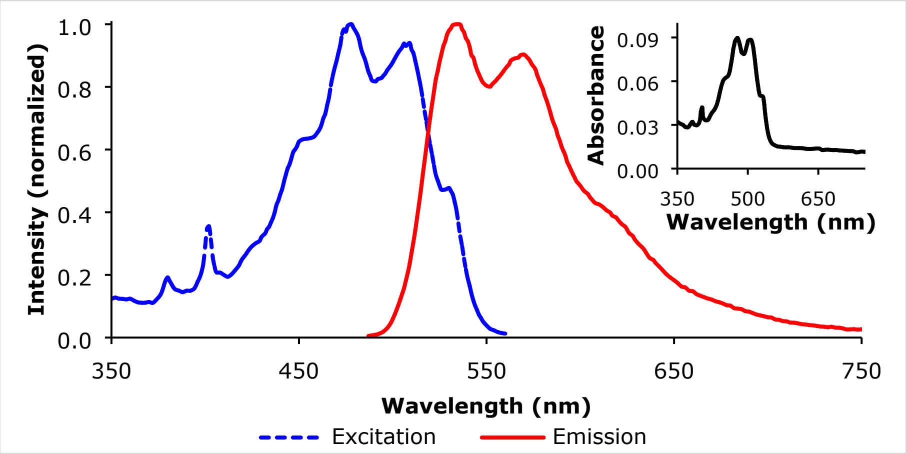

Figure S2: Excitation and emission scan for trimethylsilyl-protected **1F** in DCM. The insert shows the absorption spectrum in the same region.

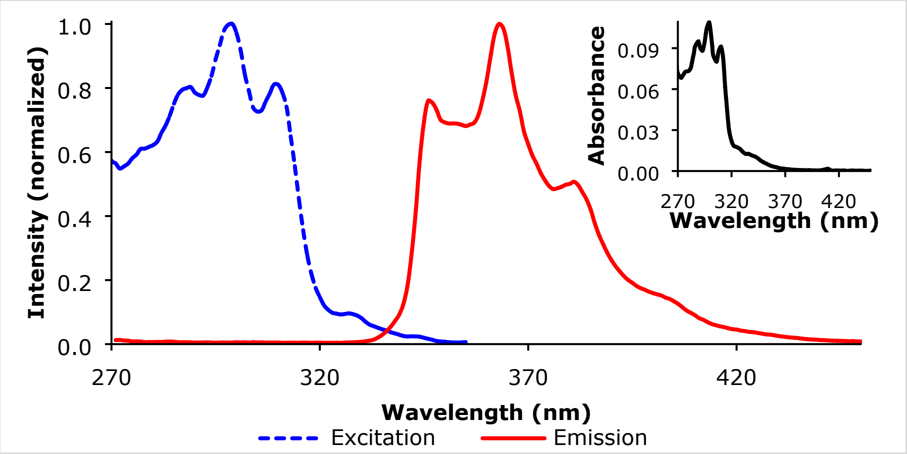

Figure S3: Excitation and emission scan for trimethylsilyl-protected **2F** in DCM. The insert shows the absorption spectrum in the same region.

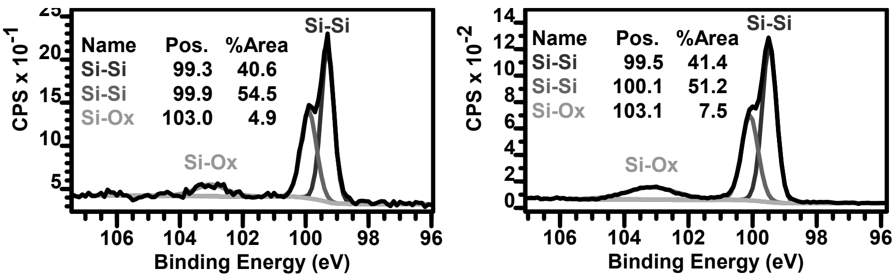

Figure S4: Si 2p narrow scans of non-backfilled surfaces functionalized with **1F** (left) and **2F** (right) after several weeks exposure to ambient atmosphere.

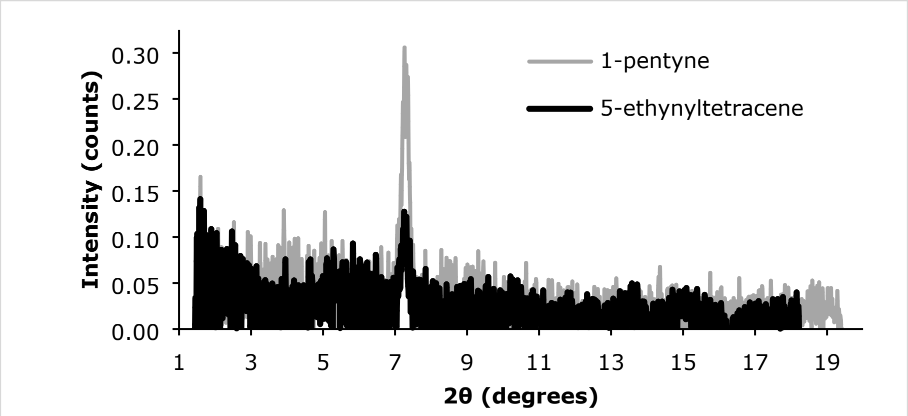

Figure S5: 2D XRD measurement on a surface functionalized with **1**. A spectrum of a surface functionalized with 1-pentyne is shown as a background spectrum.

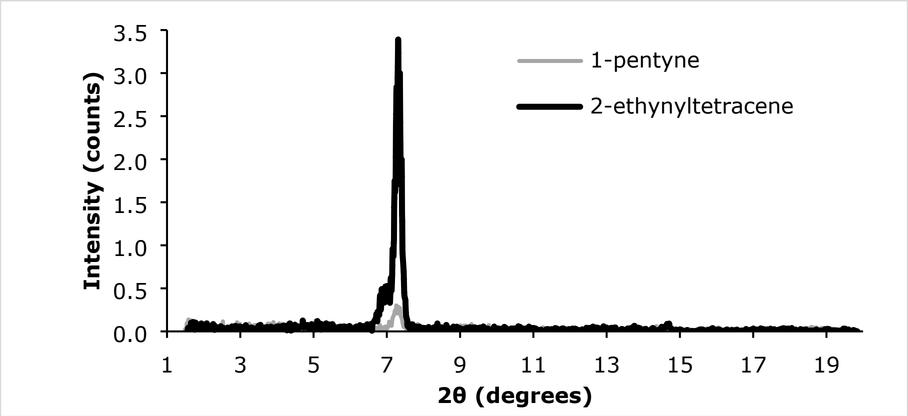

Figure S6: 2D XRD measurement on a surface functionalized with **2**. A spectrum of a surface functionalized with 1-pentyne is shown as a background spectrum.

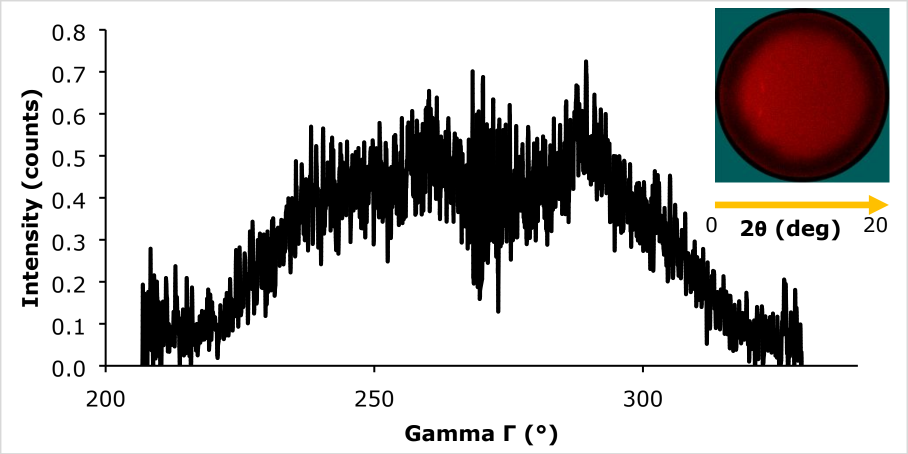

Figure S7: GIWAX spectrum of a surface functionalized with **1**, recorded at 7.3° 2θ. Data has been corrected for background signals by recording a background spectrum at a 2θ angle where no signal was present. The insert shows a picture of the surface during the GIWAX measurement, where a lighter colour indicates a higher intensity signal.

References

(1) Kulkarni, G. C.; Morales-Cruz, J. L.; Hussain, W. A.; Garvey, I. J.; Plunkett, K. N. Electron Acceptors Based on Cyclopentannulated Tetracenes. (2018), *Synlett*, **29** (19), 2572–2576.

(2) Korovina, N. V.; Das, S.; Nett, Z.; Feng, X.; Joy, J.; Haiges, R.; Krylov, A. I.; Bradforth, S. E.; Thompson, M. E. Singlet Fission in a Covalently Linked Cofacial Alkynyltetracene Dimer. (2016), *Journal of the American Chemical Society*, **138** (2), 617–627.

(3) Zhao, L.; Kaiser, R. I.; Lu, W.; Ahmed, M.; Evseev, M. M.; Bashkirov, E. K.; Azyazov, V. N.; Tönshoff, C.; Reicherter, F.; Bettinger, H. F.; Mebel, A. M. A Free-Radical Prompted Barrierless Gas-Phase Synthesis of Pentacene. (2020), *Angewandte Chemie International Edition*, **59** (28), 11334–11338.

(4) Woodward, S.; Ackermann, M.; Ahirwar, S. K.; Burroughs, L.; Garrett, M. R.; Ritchie, J.; Shine, J.; Tyril, B.; Simpson, K.; Woodward, P. Straightforward Synthesis of 2- and 2,8-Substituted Tetracenes. (2017), *Chemistry – A European Journal*, **23** (32), 7819–7824.

(5) Schwaben, J.; Münster, N.; Klues, M.; Breuer, T.; Hofmann, P.; Harms, K.; Witte, G.; Koert, U. Efficient Syntheses of Novel Fluoro-Substituted Pentacenes and Azapentacenes: Molecular and Solid-State Properties. (2015), *Chemistry – A European Journal*, **21** (39), 13758–13771.
